# Within-Day Energy Balance and Metabolic Suppression in Male Collegiate Soccer Players

**DOI:** 10.3390/nu13082644

**Published:** 2021-07-30

**Authors:** Sihyung Lee, Kuniko Moto, Seungah Han, Taewoong Oh, Motoko Taguchi

**Affiliations:** 1 Graduate School of Sport Sciences, Waseda University, Saitama 359-1192, Japan; move3210@akane.waseda.jp (S.L.); kmoto@asagi.waseda.jp (K.M.); 2 Department of Sports and Leisure Studies, Yongin University, Yongin-si 17092, Korea; gkstmddk0815@hanmail.net (S.H.); ohtw1972@gmail.com (T.O.); 3 Faculty of Sport Sciences, Waseda University, Saitama 359-1192, Japan

**Keywords:** within-day energy balance, resting energy expenditure, metabolic suppression, relative energy deficiency in sport

## Abstract

Metabolic suppression due to relative energy deficiency can cause various physiological impairments in athletes. The purpose of this study was to evaluate within-day energy balance (WDEB) and the ratio between measured and predicted resting energy expenditure (REE_ratio_) and to investigate the relationships between the markers of metabolic suppression. Ten male collegiate soccer players completed a 7-day food diary, physical activity, and heart rate records during the training and rest days. Energy intake (EI) and energy expenditure (EE) were analyzed to evaluate WDEB components. Body composition was measured using dual-energy X-ray absorptiometry (DXA), and blood sampling was conducted for hormonal analysis. The REE was measured using the Douglas bag method and predicted using the DXA-predicted method to calculate the REE_ratio_. Participants were categorized into the normal (REE_ratio_ ≥ 0.94, *n* = 5) and suppressed (REE_ratio_ < 0.94, *n* = 5) groups. There were no group differences in the components of WDEB, except diet-induced thermogenesis (DIT), but EI was significantly higher in the normal group than in the suppressed group (7-day total: 3660 ± 347 vs. 3024 ± 491 kcal/day, *p* = 0.046 and rest days: 3772 ± 463 vs. 2796 ± 800 kcal/day, *p* = 0.046). Analysis of hormonal markers of metabolic suppression only showed a significant positive association between insulin-like growth factor-1 (IGF-1) and REE_ratio_ (*r* = 0.771, *p* = 0.009). The relationships between metabolic suppression and the markers of energy deficiency were inconclusive. There are possible associations of insufficient EI and IGF-1 levels with metabolic suppression, and further study is required to understand energy deficiency in male soccer players.

## 1. Introduction

Energy balance (EB) is important for maintaining appropriate body weight and preventing the negative effects of excessive and insufficient energy. In athletes, excessive energy expenditure (EE) from training and/or matches is inevitable, but energy intake (EI) is often insufficient to achieve an appropriate energy status. The imbalanced energy status of athletes has been widely studied, and the concept of relative energy deficiency in sport (RED-S) suggested the effect of low energy availability (EA), which can result in altered health and performance in both male and female athletes [1].

EA is calculated by subtracting exercise energy expenditure (EEE) from EI and normalized to fat-free mass (FFM). Monitoring EA is important to prevent various physiological impairments, including endocrine, metabolic, and psychological problems [2], but assessing precise EA in free-living athletes is difficult because of limitations resulting from measurement errors and burden in athletes [3].

Recent studies have presented the concepts of within-day energy balance (WDEB) and the ratio between measured and predicted resting energy expenditure (REE_ratio_) to understand energy deficiency and metabolic suppression [4,5,6,7]. Assessment of WDEB measures EI and EE in 1 h intervals, and real-time changes in energy status help to identify the imbalanced energy within a day [8]. Traditional multiple-day EB and EA assessments can mask the energy deficiency by the compensation effect, while WDEB can detect the hidden energy deficiency with more physiological relevance, showing association with liver glycogen and brain glucose availability [7,9,10]. The results of previous studies on WDEB have shown associations between negative WDEB status and metabolic suppression in male and female athletes [4,7]. Reduction in REE can be interpreted as the alterations in metabolically active organs and tissues, and reduced REE has been reported in clinical energy deficiency, which is 60–80% of the predicted REE [11]. The difference between measured and predicted REE can identify the metabolic suppression by energy deficiency, and REE_ratio_ is significantly associated with the conditions related to energy deficiency in exercising women [12,13,14].

The concepts of WDEB and REE_ratio_ can provide options to monitor the energy deficiency of athletes in free-living status, which excludes the many limitations in measuring EA. However, there are limited studies on the WDEB and REE_ratio_ of male athletes, as this has been mainly studied in female participants [4,15]. To the best of our knowledge, the research by Torstveit et al. [7] is the only study to present the relationship between energy deficiency and metabolic suppression in male athletes by evaluating the WDEB and REE_ratio_. Nutritional analysis of soccer players clearly showed that there is a high risk of energy deficiency, but there is a lack of studies examining the association between energy deficiency and the markers of RED-S [16,17,18,19].

Therefore, the purpose of this study was to evaluate and analyze WDEB and REE_ratio_ in male soccer players in the free-living status and to investigate the relationship between the markers of energy deficiency and metabolic suppression.

## 2. Materials and Methods

### 2.1. Study Design

A full description of the detailed study design and methods was presented in our previous study [20]. Data were collected during the training season of October 2018. Participants visited the laboratory twice for measurements of body composition, maximal oxygen uptake, REE, and blood sampling. After the laboratory visits, participants were asked to record consecutive 7-day (5 training days + 2 rest days) food diaries and physical activity. Moreover, they were instructed to wear a heart rate (HR) sensor during training and an accelerometer from waking up until sleep except when showering, swimming, and training. The food diaries and physical activity records were checked by the researcher (S.L.) during the interviews with each participant after data collection. All participants participated in team training scheduled by the coaching staff during weekdays (3–4 h/day), and some of the participants had individual training sessions (1–2 h/day).

### 2.2. Participants

Fifteen male Korean collegiate soccer players aged 18–21 years were recruited from a local university team competing in a national university league. During the study, one participant dropped out due to personal reasons, and two participants sustained injuries. A total of 12 participants completed the data collection, but data of two participants were excluded because of non-compliance with wearing devices and absence from training resulting in incomplete data for 7 consecutive days. Data of 10 participants (mean ± SD; age 19.1 ± 0.6 years; height, 175.8 ± 5.5 cm; weight, 69.81 ± 6.14 kg) were analyzed in this study. All participants provided written informed consent after being informed of the study design and the risks of the experimental procedures. This study was approved by the Human Research Ethics Committee of Waseda University in accordance with the Declaration of Helsinki (2018-082).

### 2.3. Body Composition and Maximal Oxygen Uptake

All participants underwent a dual-energy absorptiometry (Lunar Prodigy Advance with enCORE software version 16, General Electric, Madison, WI, USA) scan to measure bone mineral density (BMD), percent body fat, fat mass, and fat-free mass (FFM).

Maximal oxygen uptake (VO_2_ max) was determined by an incremental test using a bicycle ergometer (Ergomedic 828E, Monark, Varberg, Sweden) until the participants presented the following criteria: unable to maintain 60 rpm, respiratory exchange ratio (RER) > 1.1, VO_2_ plateau, HR max (220-age), and rate of perceived exertion >19. During the test, VO_2_, carbon dioxide production (VCO_2_), and RER were measured using a breath-by-breath gas analyzer (Quark b, version 10.0, Cosmed, Rome, Italy), and HR was measured using a HR sensor and trackers (H7 and A300 fitness tracker, Polar Electro Oy, Kempele, Finland).

### 2.4. Resting Energy Expenditure and Blood Sampling

The REE of the participants was measured by indirect calorimetry using the Douglas bag technique. Participants were only allowed drinking water and instructed to avoid caffeine and alcohol intake and strenuous exercise 24 h before the measurement. The measurements were conducted between 7:00 a.m. and 9:00 a.m. after an overnight fast. Expired gas was collected for 10 min, and VO_2_ and VCO_2_ were analyzed using a gas analyzer (AE-100i, Minato Medical Science Co. Ltd., Osaka, Japan). Sample collections were continued until there was less than 5% of REE difference between the two samples, and the volume of expired air was measured using a dry gas volume meter (DC-5A, Shinagawa, Tokyo, Japan). The measured REE (REE_m_) was assessed using the Weir equation, and the mean values of the two samples with the lowest differences were used for analysis. The predicted REE (REE_p_) was calculated using the DXA-predicted method [21]. The ratio between REE_m_ and REE_p_ (REE_ratio_) was calculated as the REE_m_/REE_p_, and a participant with less than 0.94 REE_ratio_ was considered to be metabolically suppressed [5,6]. Participants were then categorized into two groups with the normal REE_ratio_ (*n* = 5) and the suppressed REE_ratio_ (*n* = 5).

After the REE measurements, blood samples were collected in the fasting state. Triiodothyronine (T_3_), cortisol, and insulin were assessed via an electrochemiluminescence immunoassay method, and insulin-like growth factor-1 (IGF-1) and growth hormone (GH) levels were assessed via a chemiluminescent immunoassay method. All blood sample analyses were conducted in a clinical laboratory (GC Labs, Yongin, Korea). Reference values were obtained from the analysis laboratory.

### 2.5. 24 h Energy Intake

Participants recorded all consumed foods and beverages for one week (5 training days + 2 rest days) using the provided cooking scale (SD-004, Tanita, Tokyo, Japan); this included the time of day and digital photographs of foods using a 15-cm ruler as a reference and of nutrition information on food packets. A registered dietitian (S.L.) checked the food records and photographs, and each participant attended an interview with the dietitian to clarify any ambiguous information. EI was analyzed using the Computer Aided Nutritional Analysis Program (CAN-Pro 5.0, The Korean Nutrition Society, Seoul, Korea) and was calculated hourly according to food records showing the time consumed. Validity of the dietary records was analyzed using the Goldberg cut-off, and all participants met the criteria for plausible reporters (EI:REE_p_ = 1.17–2.78) [22].

### 2.6. 24 h Energy Expenditure

#### 2.6.1. Resting Energy Expenditure and Sleeping Energy Expenditure

The hourly REE was calculated from REE_m_, and during the sleeping hours, sleeping energy expenditure (SEE) was calculated as 90% of REE_m_ [7]. To monitor the current metabolic status of the participants, adapted REE_m_ was used instead of unadapted REE_p_.

#### 2.6.2. Diet Induced Thermogenesis

Diet induced thermogenesis (DIT) was calculated as 10% of EI and calculated hourly after the meal or snack using the following equation: 175.9 × T × *e* ^− T/1.3^ [7,23].

#### 2.6.3. Exercise Energy Expenditure and Excessive Post-Exercise Oxygen Consumption

Participants were instructed to wear HR sensors and trackers (H7 and A300 fitness tracker, Polar Electro Oy, Kempele, Finland) during the scheduled team training and individual training. The HR data, HR-VO_2_, and HR-VCO_2_ regression equations from the VO_2_ max measurements were used to estimate VO_2_ and VCO_2_, and EE was calculated using the Weir equation [24]. The net EEE was calculated by subtracting the REE_m_ during training. Excessive post-exercise oxygen consumption (EPOC) was determined as 5% of net EEE in the first hour and 3% of net EEE in the second hour post-exercise [7,25].

#### 2.6.4. Non-Exercise Activity Thermogenesis

Non-exercise activity thermogenesis (NEAT) was determined by analyzing the data from the accelerometer (Active Style Pro HJA-750C, Omron, Kyoto, Japan) and physical activity records. The net NEAT was calculated by subtracting the REE_m_ during the activity.

### 2.7. Within-Day Energy Balance

The hourly EB was calculated using the following equation: EB = EI − total EE; REE_m_ (or SEE) + DIT + net EEE + EPOC + net NEAT, and 24 h EB was calculated as the sum of hourly EB. Each energy component was calculated according to food and physical activity records. Within-day energy deficiency (WDED) variables were determined as total hours with energy deficit (WDEB < 0 kcal), hours spent in exceeding 400 kcal of energy deficit (WDEB < −400 kcal), and the largest energy deficit in one hour [7,9,10].

### 2.8. Within-Day Energy Availability

To prevent the masking effect of EA calculation method, the hourly EA was also calculated by subtracting the net EEE from EI relative to FFM in kilograms for every hour, and 24 h EA was calculated as the sum of hourly EA [7].

### 2.9. Statistical Analysis

IBM SPSS statistics (Version 26, IBM, Somers, NY, USA) was used for statistical data analysis with a statistical significance level of *p* < 0.05. All data were tested for normality using the Shapiro–Wilk test and are presented as mean ± SD. Differences between the normal (REE_ratio_ ≥ 0.94) and suppressed (REE_ratio_ < 0.94) groups were analyzed using the independent t-test (normal distribution) and Mann–Whitney U test (non-normal distribution). The associations of REE_ratio_ and WDED variables with metabolic markers were analyzed using Pearson’s correlation (normal distribution) and Spearman’s rho (non-normal distribution).

## 3. Results

The descriptive characteristics of the 10 participants are presented in Table 1. There were no differences in the descriptive characteristics between the normal and suppressed groups.

The REE_ratio_ and WDEB characteristics with the total 7-day, training, and rest day variables are presented in Table 2, Table 3 and Table 4. There were significant differences in the REE_ratio_, REE_m_/FFM, EI, and DIT between the groups. Table 2 shows that the normal group had higher REE_ratio_ and REE_m_/FFM than the suppressed group (REE_ratio_ 1.03 ± 0.05 vs. 0.90 ± 0.04, *p* = 0.002 and REE_m_/FFM 29.4 ± 1.0 vs. 25.7 ± 1.4 kcal/kg/day, *p* = 0.001), and total 7-day EI and DIT were significantly higher in the normal group than in the suppressed group (EI 3660 ± 347 vs. 3024 ± 491 kcal/day, *p* = 0.046 and DIT 364 ± 33 vs. 301 ± 49 kcal/day, *p* = 0.043). There were no significant group differences during training days (Table 3). Table 4 shows that the rest day EI was significantly higher in the normal group than in the suppressed group (3772 ± 463 vs. 2796 ± 800 kcal/day, *p* = 0.046).

The associations between the REE_ratio_ and WDED variables and metabolic markers are presented in Table 5. The REE_ratio_ was significantly positively associated with the IGF-1 level (*r* = 0.771, *p* = 0.009). There were no other significant associations between the WDED variables and metabolic markers.

**Figure 1:**
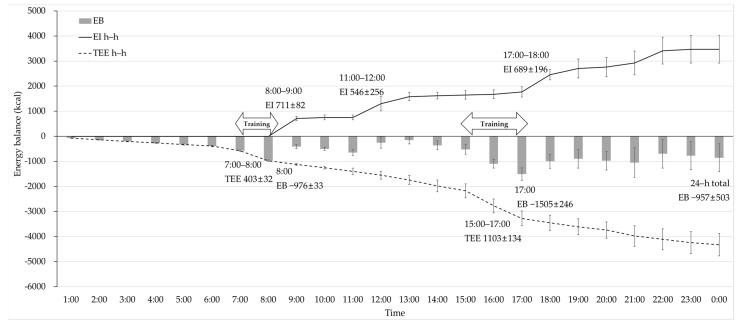
Hourly WDEB, EI, and total energy expenditure changes in the participants during the training days. EB = energy balance, EI = energy intake, TEE = total energy expenditure.

Hourly changes in the WDEB during the training days are presented in Figure 1. The lowest hourly EB was −1505 ± 246 kcal at 17:00, which was after 2 h of scheduled training spending 1103 ± 134 kcal, and after 1 h of morning training at 8:00, EB was −976 ± 33 kcal, which was spending 403 ± 32 kcal. The hourly EI was high at 8:00–9:00, 11:00–12:00, and 17:00–18:00, spending 711 ± 82 kcal, 546 ± 256 kcal, and 689 ± 196 kcal, respectively. At the end of the day, the energy deficit was −957 ± 503 kcal.

**Figure 2:**
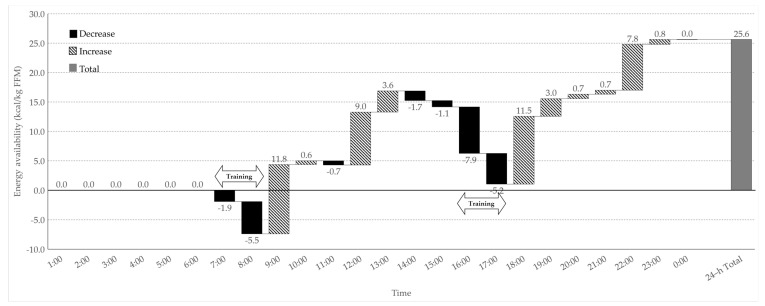
Hourly within-day energy availability change in the participants during the training days.

Hourly changes in the EA during the training days are presented in Figure 2. The EA decreased after scheduled (7:00–8:00 and 16:00–17:00) and individual (10:00–11:00) training. The hourly EA increased, except during sleeping (0:00–6:00) and training (7:00–08:00, 16:00–17:00, and 10:00–11:00) hours, and there were large increases at 8:00–9:00, 11:00–12:00, and 17:00–18:00 spending 11.8 ± 1.2, 9.0 ± 4.6, and 11.5 ± 3.9 kcal/kg FFM, respectively. The 24 h total EA was 25.6 ± 9.8 kcal/kg FFM/day.

## 4. Discussion

We analyzed the WDEB and investigated its relationship with metabolic status in Korean male collegiate soccer players. In this study, half of the participants (5 out of 10) had suppressed REE with lower total EI, rest day EI, and total DIT compared to normal participants. The REE_ratio_ had a significantly positive relationship with IGF-1 levels, but there was no association between the WDED variables and metabolic markers. Hourly changes in the WDEB during training days showed a severe energy deficit after training with insufficient compensation of EI, resulting in negative EB at the end of the day.

In this study, 50% of the participants showed metabolic suppression (REE_ratio_ < 0.94) and had significantly lower REE_ratio_ than normal participants (1.03 ± 0.05 vs. 0.90 ± 0.04, *p* = 0.002). The participants in the normal group had higher total and rest day EI than those in the suppressed group (total EI 3660 ± 347 vs. 3024 ± 491 kcal/day, *p* = 0.046, rest day EI 3772 ± 463 vs. 2796 ± 800 kcal/day, *p* = 0.046), but there were no other significant differences in the WDEB variables. In a previous study on male endurance athletes, the participants with suppressed resting metabolic ratio (RMR) had significantly lower measured RMR (kcal/h), time in severe negative EB (WDEB < −400 kcal), and larger single–hour energy deficit than normal participants, but there was no significant difference in 24 h EA and 24 h EB [7]. Another WDEB study on female endurance athletes showed that athletes with menstrual disturbance spent a longer time in WDEB < 0 kcal than eumenorrheic athletes [4]. In contrast to the findings of previous studies, we found no significant difference in the WDED variables between the normal and suppressed groups, which might be due to the different prediction equations of REE, group categorization REE_ratio_ level, and WDEB data collection methods in this study. Previous studies on the EB and EA status of athletes reported differences in the EI, EEE, and EA between the training (or match) and rest days [19,26,27,28]. Improved energy status of athletes on rest days may help to reduce the risk of physiological alterations, and studies on females showed that the addition of rest days with EI intervention could decrease the risk of menstrual dysfunction [1,29,30]. Despite the negative energy status in both groups on training days, the suppressed group had significantly lower EI on rest days than the normal group. Limited recovery of energy status during rest days may result in metabolic suppression, and further study is required to understand the importance of recovery to compensate for the severe energy deficit during training days.

A significant association was found between the REE_ratio_ and IGF-1 (*r* = 0.771, *p* = 0.009) in this study, but there was no other association between energy status and endocrine markers. The association between the endocrine markers of metabolic suppression and energy status variables has been studied to diagnose and monitor the health of athletes. Previous studies on male athletes have reported that negative EB due to undernutrition and excessive exercise results in a decrease in IGF-1 level [31,32], but low EA (15 kcal/kg FFM/day) had no effect on metabolic hormones, except leptin and insulin [33]. Recent studies on WDEB in athletes reported associations of WDED with alterations in the cortisol levels and reproductive hormones [4,7]. Additionally, the measured-to-predicted RMR ratio of exercising female participants was associated with menstrual dysfunction and low T_3_ levels [6]. We could not identify a relationship between energy deficiency and reproductive hormones, but this study supports the findings of a previous research that reported the association of REE_ratio_ with alterations in metabolic hormones. Most studies on energy deficiency have examined the effects of low EA on the health and performance of athletes. Low EA results in metabolic suppression to preserve EE required by essential processes for survival, which can cause physiological dysfunction of less critical processes; previous studies on female and male participants suggested the threshold and optimal levels of EA to prevent the adverse effects of low EA [33,34,35,36]. Many studies have applied the suggested thresholds, but the results vary depending on the participant characteristics, measurement, and analysis methods [37,38]. A recent review on the role of EA in athletes suggests the consideration of various assessment methods of energy status and of the application of the EA thresholds [39]. The REE_ratio_ and WDEB analysis may provide supporting evidence to understand the negative effect of energy deficiency on metabolic suppression and endocrine alterations, but further research is required to generate sufficient data to support application in the field of sports nutrition.

Due to severe energy deficit during training days (−957 ± 530 kcal/day), all participants showed negative 7 day EB (−561 ± 529 kcal/day), despite positive EB during rest days (429 ± 693 kcal/day). Analysis of WDEB during training days clearly showed the most significant energy deficit after the scheduled team training (−1505 ± 246 kcal, at 17:00). Energy status was slightly improved after EI from scheduled dormitory meals and snacks at the university cafeteria, but it could not fully compensate for excessive EE from the training sessions, resulting in negative 24 h total EB and 25.6 ± 9.8 kcal/kg FFM of 24-h total EA. A previous study on the hourly energy status of female collegiate soccer players presented negative 24 h EB [40], which is in accordance with the results of this study. Additionally, we analyzed the hourly energy status to understand the reason for the severe energy deficit and to provide possible suggestions for the improvement of energy status during training days. Previous studies on seasonal (training, match, and rest) and daily energy status of soccer players showed negative EB and decreased EA during the training and match seasons [17,18], with significantly lower EA during heavy training and match days than during rest days [19]. Studies on the daily distribution of EI and macronutrients in elite male soccer players have suggested the importance of collecting meal distribution data that can be related to training adaptations, performance, and health [41,42]. Moreover, the importance of diet and training periodization has been widely studied, and the interaction between nutrition and exercise has been emphasized to enhance physiological adaptations and exercise capacity [43]. We observed hourly changes in energy status in free-living athletes; this provided the specific time and reason for the energy deficit during the day with the amount of energy required to prevent a negative energy state at the end of the day. It can help provide the optimal nutritional and training strategy for athletes; therefore, the effects of various characteristics of athletes and training environments on WDEB should be considered in future studies.

To our knowledge, this is the first study to analyze the WDEB variables and metabolic status in male collegiate soccer players, but there are several limitations to the study design and methodologies. The small sample size of this study decreases the statistical power of analysis and limits the generalizability of the findings. Due to the relatively short period of the experiment, the time point of the blood analysis was restricted. Data collection of the 7-day WDEB variables relied on self-reported measurements in a free-living status, which could have resulted in under- and over-reporting of EI and the inaccuracy of activity records.

## 5. Conclusions

This study aimed to analyze the WDEB variables and REE_ratio_ of male athletes as the markers of energy deficiency and to investigate the relationship between energy deficiency and metabolic suppression. A significantly lower rest day EI in the metabolically suppressed group (REE_ratio_ < 0.94) and a positive association between REE_ratio_ and IGF-1 level were observed. Additionally, WDEB analysis of training days showed a severe energy deficit after training hours with insufficient energy consumption for daily EB. In conclusion, metabolic suppression can be related to insufficient EI during rest days, which could not compensate for the severe energy deficit during training days, and IGF-1 level in male soccer players. Monitoring REE_ratio_ and WDEB can help provide practical advice and support to prevent the negative effects of energy deficiency in athletes. Future studies should include a larger number of participants in different types of sports and should consider more accurate methodologies for the collection of EI and EE data.

## Figures and Tables

**Table 1:** Descriptive characteristics of the participants.

	Total	Normal (*n* = 5)	Suppressed (*n* = 5)	*p*-Value
Age (years)	19.1 ± 0.6	19.2 ± 0.8	19.0 ± 0.0	0.519
Height (cm)	175.8 ± 5.5	173.0 ± 1.9	178.7 ± 6.7	0.135
Weight (kg)	69.81 ± 6.14	67.66 ± 4.41	71.96 ± 7.34	0.294
BMI (kg/m^2^)	22.5 ± 1.3	22.6 ± 1.4	22.5 ± 1.4	0.930
BMD (g/cm^2^)	1.378 ± 0.081	1.391 ± 0.070	1.366 ± 0.097	0.655
Z-score	1.6 ± 0.8	1.8 ± 0.8	1.4 ± 0.8	0.416
Body fat (kg)	9.4 ± 2.4	9.2 ± 2.0	9.6 ± 3.0	0.831
Body fat (%)	13.3 ± 2.4	13.5 ± 2.3	13.1 ± 2.7	0.819
FFM (kg)	60.4 ± 4.3	58.5 ± 3.0	62.4 ± 4.7	0.159
FFM (%)	86.7 ± 2.4	86.5 ± 2.3	86.9 ± 2.7	0.819
VO_2_ max (mL/kg/min)	55.6 ± 6.0	53.0 ± 4.6	58.2 ± 6.5	0.180

Note: BMI = body mass index, BMD = bone mineral density, FFM = fat-free mass; data are presented as mean ± SD.

**Table 2:** REE_ratio_, EI, EE components, and WDED variables of the participants during total 7 day in total.

	Total	Normal (*n* = 5)	Suppressed (*n* = 5)	*p*-Value
REE_ratio_ (REE_m_/REE_p_)	0.96 ± 0.08	1.03 ± 0.05	0.90 ± 0.04	0.002 *
REE_m_/FFM (kcal/kg/day)	27.6 ± 2.3	29.4 ± 1.0	25.7 ± 1.4	0.001 *
EI (kcal/day)	3342 ± 522	3660 ± 347	3024 ± 491	0.046 *
DIT (kcal/day)	332 ± 52	364 ± 33	301 ± 49	0.043 *
Net EEE (kcal/day)	1391 ± 310	1458 ± 420	1324 ± 168	0.537
EPOC (kcal/day)	125 ± 28	132 ± 37	119 ± 17	0.513
Net NEAT (kcal/day)	456 ± 100	469 ± 70	443 ± 131	0.702
REE_h_ (kcal/day)	1057 ± 141	1131 ± 150	982 ± 93	0.096
SEE (kcal/day)	542 ± 42	531 ± 32	552 ± 52	0.473
TEE (kcal/day)	3903 ± 415	4085 ± 434	3721 ± 342	0.179
24-h EB (kcal)	−561 ± 529	−426 ± 621	−697 ± 444	0.450
24-h EA (kcal/kg FFM)	32.7 ± 11.0	37.8 ± 11.8	27.5 ± 8.0	0.146
WDEB < 0 kcal (h/day)	20.2 ± 1.8	20.0 ± 1.9	20.4 ± 1.9	1.000
WDEB < −400 kcal (h/day)	11.9 ± 1.9	12.0 ± 1.9	11.8 ± 2.0	0.876
Largest hourly deficit (kcal)	−1509 ± 243	−1572 ± 277	−1446 ± 214	0.446

Note: REE_m_ = measured resting energy expenditure, REE_p_ = predicted resting energy expenditure, REE_m_/FFM = ratio between measured resting energy expenditure and fat-free mass, EI = energy intake, DIT = diet induced thermogenesis, EEE = exercise energy expenditure, EPOC = excess post-exercise oxygen consumption, NEAT = non-exercise activity thermogenesis, REE_h_ = hourly resting energy expenditure, SEE = sleeping energy expenditure, EB = energy balance, EA = energy availability, WDEB = within-day energy balance; data are presented as mean ± SD. * *p* < 0.05. significant between-group difference.

**Table 3:** EI, EE components, and WDED variables of the participants during training days.

	Total	Normal (*n* = 5)	Suppressed (*n* = 5)	*p*-Value
EI (kcal/day)	3365 ± 479	3615 ± 428	3116 ± 422	0.100
DIT (kcal/day)	332 ± 48	359 ± 42	304 ± 40	0.068
Net EEE (kcal/day)	1831 ± 357	1903 ± 508	1759 ± 124	0.567
EPOC (kcal/day)	165 ± 32	172 ± 45	158 ± 13	0.516
Net NEAT (kcal/day)	394 ± 114	393 ± 63	396 ± 160	0.974
REE_h_ (kcal/day)	1081 ± 133	1146 ± 151	1017 ± 81	0.132
SEE (kcal/day)	519 ± 47	518 ± 36	520 ± 60	0.966
TEE (kcal/day)	4323 ± 469	4491 ± 558	4154 ± 335	0.280
24-h EB (kcal)	−957 ± 530	−876 ± 701	−1038 ± 353	0.657
24-h EA (kcal/kg FFM)	25.7 ± 10.4	29.6 ± 12.8	21.9 ± 6.6	0.265
WDEB < 0 kcal (h/day)	21.3 ± 1.3	21.0 ± 1.6	21.6 ± 1.1	0.511
WDEB < −400 kcal (h/day)	12.7 ± 1.6	12.8 ± 1.9	12.6 ± 1.3	0.854
Largest hourly deficit (kcal)	−1718 ± 278	−1792 ± 348	−1644 ± 197	0.434

Note: EI = energy intake, DIT = diet induced thermogenesis, EEE = exercise energy expenditure, EPOC = excess post-exercise oxygen consumption, NEAT = non-exercise activity thermogenesis, REE_h_ = hourly resting energy expenditure, SEE = sleeping energy expenditure, EB = energy balance, EA = energy availability, WDEB = within-day energy balance; data are presented as mean ± SD.

**Table 4:** EI, EE components, and WDED variables of the participants during rest days.

	Total	Normal (*n* = 5)	Suppressed (*n* = 5)	*p* Value
EI (kcal/day)	3284 ± 803	3772 ± 463	2796 ± 800	0.046 *
DIT (kcal/day)	335 ± 85	378 ± 47	292 ± 96	0.111
Net EEE (kcal/day)	291 ± 292	345 ± 279	238 ± 327	0.666
EPOC (kcal/day)	26 ± 26	31 ± 24	21 ± 29	0.666
Net NEAT (kcal/day)	609 ± 138	658 ± 142	560 ± 128	0.286
REE_h_ (kcal/day)	995 ± 172	1094 ± 156	895 ± 131	0.061
SEE (kcal/day)	598 ± 61	564 ± 46	632 ± 59	0.076
TEE (kcal/day)	2855 ± 398	3071 ± 268	2639 ± 410	0.084
24-h EB (kcal)	429 ± 693	701 ± 602	157 ± 730	0.234
24-h EA (kcal/kg FFM)	50.1 ± 16.0	58.8 ± 13.3	41.5 ± 14.5	0.075
WDEB < 0 kcal (h/day)	17.8 ± 4.1	17.6 ± 3.6	18.0 ± 4.9	0.888
WDEB < −400 kcal (h/day)	9.8 ± 3.0	9.8 ± 2.8	9.8 ± 3.6	1.000
Largest hourly deficit (kcal)	−986 ± 239	−1021 ± 231	−951 ± 269	0.668

Note. EI = energy intake, DIT = diet induced thermogenesis, EEE = exercise energy expenditure, EPOC = excess post-exercise oxygen consumption, NEAT = non-exercise activity thermogenesis, REE_h_ = hourly resting energy expenditure, SEE = sleeping energy expenditure, EB = energy balance, EA = energy availability, WDEB = within-day energy balance; data are presented as mean ± SD. * *p* < 0.05. significant between-group difference.

**Table 5:** Associations of REE_ratio_ and WDEB variables with metabolic markers in the participants.

	REE_ratio_ (REE_m_/REE_p_)		REE_m_/FFM		WDEB < 0 kcal (h/day)		WDEB < −400 kcal (h/day)		Largest Hourly Deficit (kcal)		24-h EB (kcal)		24 h EA (kcal/kg FFM)	
	*r*	*p*-Value	*r*	*p*-Value	*r*	*p*-Value	*r*	*p*-Value	*r*	*p*-Value	*r*	*p*-Value	*r*	*p*-Value
T_3_	0.162	0.655	0.190	0.599	0.532	0.113	0.487	0.153	−0.564	0.090	−0.456	0.185	−0.234	0.514
Cortisol	0.144	0.691	0.208	0.565	−0.426	0.219	−0.498	0.143	0.206	0.568	0.188	0.602	0.208	0.564
IGF-1	0.771	0.009 *	0.590	0.072	0.106	0.771	0.223	0.536	−0.356	0.313	0.222	0.538	0.381	0.277
GH	0.566	0.088	0.509	0.133	0.275	0.442	0.475	0.165	−0.448	0.194	−0.117	0.748	−0.055	0.880
Insulin	0.151	0.678	0.046	0.899	0.043	0.906	0.108	0.767	-0.102	0.778	0.058	0.874	0.201	0.577
Testosterone	−0.552	0.098	−0.439	0.205	−0.029	0.938	−0.173	0.632	0.497	0.144	−0.067	0.853	−0.287	0.421
Leptin	0.308	0.387	0.293	0.410	0.197	0.585	0.238	0.508	−0.242	0.501	−0.189	0.601	−0.062	0.866

Note: REE_m_ = measured resting energy expenditure, REE_p_ = predicted resting energy expenditure, REE_m_/FFM = ratio between measured resting energy expenditure and fat-free mass, T_3_ = triiodothyronine, IGF-1 = insulin-like growth factor 1, GH = growth hormone; * *p* < 0.05, significant association between variables.
